# The Challenge of Human Spermatozoa Proteome: A Systematic Review

**Published:** 2017

**Authors:** Kambiz Gilany, Arash Minai-Tehrani, Mehdi Amini, Niloofar Agharezaee, Babak Arjmand

**Affiliations:** 1- Reproductive Biotechnology Research Center, Avicenna Research Institute, ACECR, Tehran, Iran; 2- Metabolomics and Genomics Research Center, Endocrinology and Metabolism Molecular Cellular Sciences Institute, Tehran University of Medical Sciences, Tehran, Iran; 3- Nanobiotechnology Research Center, Avicenna Research Institute, ACECR, Tehran, Iran; 4- Department of Genetics, Tehran Medical Sciences Branch, Islamic Azad University, Tehran, Iran; 5- Cell Therapy and Regenerative Medicine Research Center, Endocrinology and Metabolism Molecular Cellular Sciences Institute, Tehran University of Medical Sciences, Tehran, Iran

**Keywords:** Human, Proteome, Proteomics, Spermatozoa, Y chromosome

## Abstract

Currently, there are 20,197 human protein-coding genes in the most expertly curated database (UniProtKB/Swiss-Pro). Big efforts have been made by the international consortium, the Chromosome-Centric Human Proteome Project (C-HPP) and independent researchers, to map human proteome. In brief, anno 2017 the human proteome was outlined. The male factor contributes to 50% of infertility in couples. However, there are limited human spermatozoa proteomic studies. Firstly, the development of the mapping of the human spermatozoa was analyzed. The human spermatozoa have been used as a model for missing proteins. It has been shown that human spermatozoa are excellent sources for finding missing proteins. Y chromosome proteome mapping is led by Iran. However, it seems that it is extremely challenging to map the human spermatozoa Y chromosome proteins based on current mass spectrometry-based proteomics technology. Post-translation modifications (PTMs) of human spermatozoa proteome are the most unexplored area and currently the exact role of PTMs in male infertility is unknown. Additionally, the clinical human spermatozoa proteomic analysis, anno 2017 was done in this study.

## Introduction

### Anno 2017; from proteome to proteoform:

There was a revelation obtained from human genome project results showing a smaller number of analyzed genes that was approximately 20,300 rather than ∼100,000 (1). This finding determined that the complication in our biological system can be due to variation at the level of protein rather than a large number of distinct genes (2). The diversity among well related protein molecules which are chemically different can be driven from variation within populaces, cell and tissue and their subcellular localization. The intricacy of allelic variations, alternative splicing of RNA transcripts and post-translational modifications can be due to DNA, RNA and protein structures and levels. These machineries build distinct protein molecules which are able to affect cell signaling, gene regulation and activation of protein complexes. Although the complexity of protein structures was first identified by using two-dimensional gel electrophoresis, novel proteomic technologies have been demonstrated to develop the decisive architectures of proteome (3, 4). The word proteome was first introduced in 1995 by Wilkins. The term has become quite popular due to its simple and easy definition (5). The global protein analysis idea somehow is not new. It was first appeared in late 1970s (6). Nowadays, this scientific field is called proteomics, which is the study of the large-scale number of proteins expressed in a cell, tissue or biological fluids (7, 8). In general, proteomics is divided in five central pillars: mass spectrometry-based proteomics, array-based proteomics, structural proteomics, clinical proteomics and informatics (9).

Mass spectrometry has developed as a key platform for proteomic analyses, with two contrasting approaches which are bottom-up and top-down proteomics. In the bottom-up approach, typically the enzyme trypsin is used to digest the proteome to small peptides. The complex peptide mixture is separated and analyzed by liquid chromatography tandem-mass spectrometry (LC-MS/MS) (10, 11). In top-down proteomics, the proteins of the proteome in an unbroken fashion are directly separated and fragmented by LC-MS/MS. The top-down approach has been reported to provide the most useful data for accurate identification and characterization of molecular composition (12, 13). On the other hand, executing bottom-up approach can be difficult due to the complexity of produced results and limitation of technical knowledge.

MS-based proteomics is in constant movement and new terms (such as proteoform), new instrumentation and software are under development which help to identify more proteins (14). The newest term, proteoform is defined as all different molecular forms in which the protein product of a single gene can be found, encompassing all forms of genetic variation, alternative splicing of RNA transcripts, and post-translational modifications (PTMs) (15, 16).

### First maps of the human proteome:

It has been indicated that mass spectrometry was able to develop the analysis of human proteome in a way which is comparable to influence of next-generation sequencing on genomics and analysis of transcriptomics (7, 17, 18). The international consortium for the Chromosome-Centric Human Proteome Project (C-HPP) was established in 2011. The propose of C-HHP is to identify and characterize each of the 20,300 human protein coding genes including single amino acid polymorphisms (SAPs), splice variant isoforms and post-translational modifications (PTMs) (19–21). The guidelines for C-HPP are set, and each of international teams has selected a specific chromosome (22, 23).

More than 150 posters and papers have been published by C-HPP since 2013 (www.thehpp.org). Using combined MS-based proteomics and antibody technology followed by bioinformatics, less than 14,000 protein-coding genes have been identified (24).

Based on two independent studies from HUPO organization, draft maps of human proteome were published in 2014 using MS-based proteomics platform. They were able to identify 17,294 and 19,629 protein encoding genes, respectively (25, 26). The draft maps of the human proteomics were in larger scale than the multinational Human Proteome Project effort. In their studies, Kim et al. and Wilhelm et al. have used different healthy biological samples from human including testis. However, none of them used the human spermatozoa to map the human proteome. Finalizing the human proteome is more challenging than genome. It will have much more greater impact on understanding human diseases. The first analysis of the portray of human proteome clearly shows this challenge (27). Ezkurdia et al. analyzed the data from the human proteome and showed that Kim et al. and Wilhelm et al. have overestimated their identification of proteins in case of trans-membrane proteins. Furthermore, they showed both studies have an abundance of poor spectra, low-scoring peptide-spectrum matches and incorrectly identified proteins (28). Additionally, a reanalyzation of Kim et al.’s study by HUPO team showed that they could identify only 11,000 not 17,000 genes as they hypothesized (29). However, despite these critical analyses of the first mapping of the human proteome, it does not undervalue these studies, since they have more than 500 citations two years after publication.

MS-based proteomics technology has improved during years and it has become a useful biomedical research instrument. MS-based technology can help in diagnosis of disease-related mutations and biomarkers. However, lack of genomic data in infertility or other diseases associated with proteomics data is still an undetermined issue.

The main goal of the medical proteomics is to find disease biomarkers including proteins or peptides that are specific to a disease. This goal has not been reached yet. However, Human Proteome Project and other independent studies are inspiring concerning medical proteomics (30–35).

### The size of the human spermatozoa proteome:

It has been reported that the recognition and evaluation of expressed proteins in cells and tissues could develop a better approach to understand the cell dynamics and tissue purposes in a variety of fields (36). Human spermatozoa can provide optimal cells to be investigated from a proteomic perspective because they do not represent physiologically active transcription and translation. As such, proteomics has the potential to transform our understanding of the workings of the mature cell. Such a leap in knowledge is necessary as spermatozoa are very specialized cells (37).

**Figure 1.**
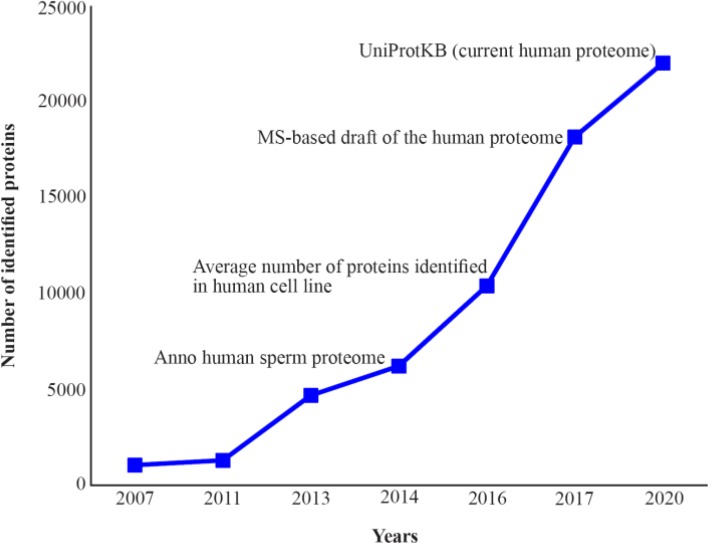
The human spermatozoa proteome mapping development compared to the human cell line proteome and the current known human proteome

After a moderate initiation for the proteome analysis of the human spermatozoa, a quick development has been created in the last couples of years (Figure 1) (38–41).

An earlier attempt to map the human spermatozoa proteome was published in 2005 (42). The authors claimed that they have identified over 1,700 human spermatozoa proteins, however, no specified list of correlated proteins has been declared by them (42). In 2007, the first large scale analysis of the human spermatozoa proteome was published with a protein list that identified 1,053 proteins (38). The first attempt to organize and catalogue the human spermatozoa proteome was done in 2011 by the authors (39). The collection of 1,300 proteins was reported. Following development of MS-based proteomics technology, the human spermatozoa proteome was further subjected to proteomic analysis. Wang et al. identified 4,675 human spermatozoa proteins, of which 227 were testis-specific (40). The latest investigation that catalogued the human spermatozoa proteome was done by Amaral et al. (41). They were able to collect 6,198 unique human spermatozoa proteins. Finally, the question is: how big is the size of the human spermatozoa proteome?

For a long, due to transcriptional inactivity of sperm cells, human spermatozoa proteome is believed to be restricted to a couple of thousand proteins (37, 39). By looking at the development of the human spermatozoa proteome analysis during the last years, it seems that the depth of understanding in human spermatozoa proteome has not been reached yet (Figure 1). It is reported that MS-based proteomics technology is not limited by sensitivity. It is rather limited by dynamic range and effective sequencing speed (43). Furthermore, it has been shown that human cell line proteome analysis by the MS-based technology has reached saturation level for quantification and identification in 2011 (Average number of protein in the human cell line, figure 1) although an improved resolution and sequencing speed of mass analyzer was achieved (44).

MS-based proteomics technology has further developed leading to have deeper view of the human proteome. It seems that by the development of MS-based proteomics technology and software, it becomes easier to analyze the current human proteome (MS-based draft of the human proteome and UniProtKB current human proteome, figure 1) (25, 26, 45). The current size of the human proteome is based on protein-coding genes (21,931 proteins), while the isoforms, PTM or alternative splicing are not included in the mentioned version. The Proteomics DB database, which is the outcome of the first draft of human proteome has 86,771 isoforms (46). Another database, which solely developed for the mass spectrometry (MS) identification of human proteins, is neXtProt. This database demonstrates proteins existence, their related isoforms, post-translational modifications as well as subcellular localization (47). Almost 400 proteins are found in the neXtProt by searching the term of “spermatozoa”.

One of the challenges of the proteomics study is to analyze the proteome quantitatively. Estimating protein concentration in a cell is significant. Understanding cell biology depends on the knowledge of the cellular protein quantities. For example, system biology approach which describes behavior of a cell depends on the knowledge of protein copy number per cell whereas estimation of absolute protein concentration of human protein is technically challenging and limited (48, 49). It is believed that no study has used quantitative tandem MS strategy to estimate the cellular concentration of the human spermatozoa proteome yet.

Several researchers in a variety of studies have used the bottom-up approach for proteomic analysis of the human spermatozoa. Peptide Atlas has been reported as a database that is under investigation for gathering peptides in MS-based proteomics of bottom-up approaches (50). It explains the genome via peptide identification of proteins. One of the interesting points of Peptide Atlas is collection of proteomic analysis for the male reproductive system; however, there are no studies regarding the human spermatozoa by now, to the best of our knowledge.

The analysis of human spermatozoa proteome is becoming more proteomic. However, the most recent proteomics technology approach which is top-down proteomics has not been well studied yet. It would be interesting to investigate whether unknown proteins or missing proteins (see below) of the human spermatozoa proteome can be identified or not using top-down proteomics.

### Missing proteins:

The human gene product variety should not be miscalculated according to the alternative mRNA splicing, post-translational modifications (PTMs) and polymorphism of single amino acid. To the best of our knowledge, no body exactly knows how many proteins are expressed in the ∼230 cell types that build our body while between 1 to 2 million proteoforms have been recommended (39, 51).

Among ∼20,197 human protein-coding genes, about 3834 (10%) of them lack any experimental evidence at the protein level. These proteins are named “missing proteins” (51). There are many reasons why these proteins lack an evidence of experimental protein expression in the data bases *e.g*. UniProtKB or neXtProt. Lack of the evidence for protein expression level has been reported to be due to: 1) Protein specificity for each organ; 2) Expression of specified proteins in the early development stage, particularly embryonic and fetal; 3) Being below our current limits for detection of protein expression which itself can be due to short rates of synthesis or rapid degradation; and 4) Expression of proteins under stress condition (23, 52, 53).

A great number of proteins are identified by the first draft of the human proteome using MS-based proteomics technology. However, it is clear that missing proteins can be considered as one of the main challenges for the future work. MS-based proteomics is not able to identify all proteins, *e.g*. Neuroglobin, a protein that needs to be identified by in-depth research lab, and cannot be detected by current MS-based proteomics technology (54). That can be due, at least in *part*, to the detection limits of mass spectrometry.

The ProteomicsDB database does include a section, “Adopt a protein”, which calls protein experts in the world to fill the gap in the human proteome missing proteins (46).

The C-HPP has provided a scheme to investigate the information associated with the missing proteins (55, 56). One of the exciting studies, published in the annual report of C-HPP, was the use of human spermatozoa as a model for detecting missing proteins (56). The argumentation to use the human spermatozoa for detecting the missing proteins is that the human spermatozoa proteins are cell specific. Surprisingly, the authors were able to identify 89 of the missing proteins in the human spermatozoa. They showed that the genes of these proteins were located on 20 different chromosomes. The chromosomes that do not carry the genes encoding any of these proteins were 21, 22 and Y (57).

### Y-chromosome:

In the Chromosome-centric Human Proteome Project (C-HPP), the proteome mapping of the human Y chromosome is considered to be conducted by Iran (58). The human Y chromosome is a type of sex chromosome that exists in male mammalian species basically. The human Y chromosome is about three times smaller than the human X chromosome, and its male sex-determining function is exclusively located on the short arm. Male sex determination has been reported as an outcome of gonadal sex purpose during embryonic development. In the presence of the human Y chromosome, the embryonic gonads turn into testes, however, in the absence of human Y chromosome, the gonads develop into ovaries (59, 60). It has been demonstrated that deletions or mutations, particularly in the long arm of human Y chromosome, may lead to male infertility, and also influence the reproductive performances of related sons (61–63). In relation to C-HPP, Jangravi demonstrated a current revise of the malespecific region (MSY) in the human Y chromosome protein-encoding genes. The human Y chromosome proteins were analyzed corresponding to each disease. They also indicated protein-protein interactions and post-translational modifications of protein-coding genes in the MSY (64). Most recently, Rengaraj et al. analyzed the human Y chromosome-encoded proteins (66 Y chromosome-encoded proteins retrieved from NCBI database), their related pathways, and their related interactions using bioinformatics tools (65). It is very important to understand the UniProtKB database that shows only 47 human Y chromosome-encoded proteins which have evidence on protein level (http://www.uniprot.org/docs/humchry). Regarding neXtProt and PeptideAtlas databases, the retrieved human Y chromosome-encoded proteins showed 44 and 40, respectively (65). The MS-based proteomics draft of the human proteome conducted by Wilhelm et al. showed a 57% coverage of the human Y chromosome-encoded proteins (46).

The protein pathways analysis of the 66 proteins encoded by human Y chromosome demonstrated 4 major pathways, including cell signaling pathways, receptor signaling pathways, cellular processes, and metabolic pathways (65).

An analysis of the current catalogue of the human spermatozoa proteome for human Y chromosome-encoded proteins retrieved from UniProtKB database determined the following encoded Y chromosome proteins as detected by current MS-based proteomics technology including ATP-dependent RNA helicase (O15523), Heat shock transcription factor, Y-linked (Q96LI6), Protocadherin-11 Y-linked precursor (Q9BZA8), 40S ribosomal protein S4, Y isoform 1 (P22090), Testisspecific Y-encoded protein 1 (Q01534) and ubiquitin carboxyl-terminal hydrolase FAF-Y (O00507). However, these numbers of proteins cover only ∼10% of the current human Y chromosome-encoded proteins.

The protein abundance can be roughly estimated by MS using the number of identified unique peptides of a protein (66). The six identified human Y chromosome-encoded proteins in the human spermatozoa proteome are identified with the following number of unique peptides: O15523 (4), Q96LI6 (4), P22090 (4), Q01534 (2), O00507 (3), and Q9BZA8 (Not reported). It is clear that human Y chromosome-encoded proteins are not highly expressed proteins even in the human spermatozoa cell based on the number of the unique peptides identified from the mentioned proteins.

**Figure 2.**
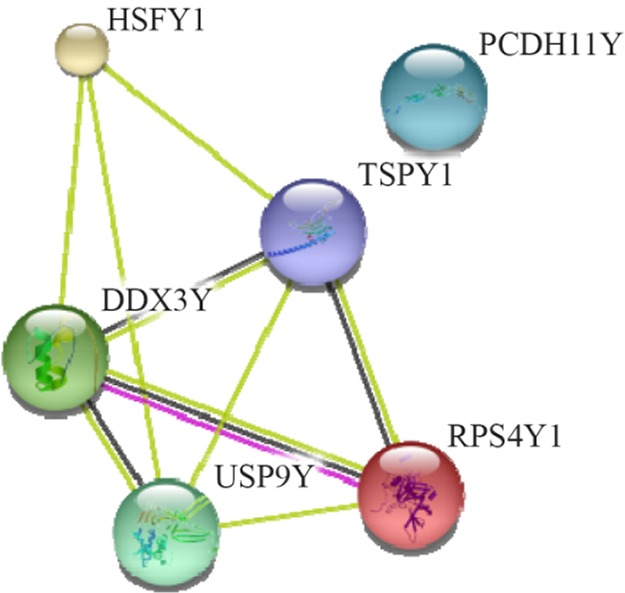
Interactions among the six human Y chromosome-encoded proteins in the human spermatozoa cell. A medium confidence view (score 0.4) of the interaction among human Y chromosome-encoded proteins was prepared using the STRING database program. Thicker lines represent stronger associations, and thinner lines represent medium associations. The UniportKB associate code and corresponding gene code are O1523 (DDX3Y), P22090 (RPS4Y1), Q96LI6 (HSFY1), Q01534 (TSPY1), O00507 (USP9Y), and Q9BZA8 (PCDH11Y)

These six human Y chromosome-encoded proteins in the human spermatozoa were analyzed for protein-protein interactions using STRING database (Figure 2) showing that in a medium confidence search (score 0.4), there is a strong interaction between O1523 (DDX3Y) and P22090 (RPS4Y1) in the human spermatozoa cell (67).

To determine the cellular pathways involving these six human Y chromosome-encoded proteins in the human spermatozoa, the Reactome pathway knowledge base database was searched (68). The six human Y chromosome-encoded proteins activate the following pathways in the human spermatozoa cell including gene expression, metabolism of proteins and signal transduction.

### Post-translational modifications in the human spermatozoa:

Spermatozoa can be considered as an ideal model for investigation of post-translational modifications since the transcriptional and translational activities are almost inactive (69, 70). It has been reported that spermatozoa functions can be mostly regulated at the protein level while its post-translational modifications (PTMs) are particularly vital. Regulating spermatozoa functions such as maturation and acquisition of fertilizing potential can be affected by PTMs on existing proteins (71).

It is very complicated to understand which PTMs are the most frequent inside the cells. Recently, UniProt database collected 307 diverse types of PTM (http://www.uniprot.org/docs/ptmlist). PTM leads to a change in total mass of the related protein and can alter the residue nature. Though the current proteome-wide statistics analysis of UniProtKB database for experimental PTMs shows the following PTMs are dominated by Phosphorylation, Acetylation, N-linked glycosylation, Amidation and Hydroxylation, the putative PTMs are first dominated by N-linked glycosylation (72).

There is a significant challenge and a required expertise in the large-scale MS-based PTMs proteomics analysis compared to conventional MS-based proteome analysis is needed.

Therefore, there is only a handful of large-scale MS-based proteomics studies of the human spermatozoa PTMs. These studies have focused on the phopshoproteome, N-linked glycoproteome, acetylproteome and ubiquitination (71, 73–75).

The full potential of large-scale MS-based proteomics technology in order to better understand PTMs functions in the human spermatozoa is not well defined. To the best of our knowledge, the only study that used high-throughput technology was carried out by Ficarro et al. They showed that the phosphorylation plays an important role and specifically valosin-containing protein was phosphorylated during capacitation. However, phosphorylated sites of this protein were not identified (74).

This paper was not about the PTM function in the human spermatoza. The purpose was just to attract attention to PTM study regarding the spermatozoa since there are limited studies avialabe related to this subject.

### Proteomic analysis of spermatozoa:

Semen analysis screening information might indicate male infertility factor while, not reflecting reproductive potential constantly (76). Therefore, screen of sperm DNA damage and oxidative stress can be recommended to forecast reproduction (77–82).

The global protein analysis/proteomics has been investigated for more than 40 years. However, only during the last 10 years, the studies regarding male infertility and spermatozoa have gained momentum (Table 1).

**Table 1.** Proteomics studies of the human spermatozoa

**Proteomics methodologies**	**Type of patients/studies**	**Outcome**	**Year (Reference)**
**2DE, in-gel digestion, LC-MS/MS and MALDI-TOF MS**	To compare the sperm protein expression profile (proteome map) from a patient who experienced failed fertilization at IVF with fertile controls	First proteome comparison of different qualities of sperm. Identification of 4 proteins differentially expressed	2004 (83)
**2DE, in-gel digestion MALDI-TOF MS**	To compare sperm protein expression profiles in asthenozoospermic patients with that of normozoospermic donors	Identification of 10 differentially expressed proteins	2007 (84)
**2DE, in-gel digestion MALDI-TOF MS**	A comparison of asthenozoospermic sperm proteins to the fertile controls	Identification of 17 differentially expressed proteins	2008 (85)
**2DE, in-gel digestion MALDI-TOF MS**	Asthenozoospermic sperm of patients were compared to fertile controls	Identification of 12 differentially phosphorylated proteins	2009 (86)
**2DE-DIGE, in-gel digestion LC-MS/MS and MALDI-TOF MS**	The spermatic proteomic profiles of patients, with a complete failure of fertilization and no spermatozoa bound to the zona pellucida, compared to controls	Identification of 12 differentially expressed proteins	2009 (87)
**2DE-DIGE, in-gel digestion MALDI-TOF/TOF-MS**	To investigate the differences in protein expression between human round-headed and normal spermatozoa	Identification of 35 proteins differentially in round-headed sperm compared with normal sperm	2009 (88)
**2DE, in-gel digestion MALDI-TOF MS and MS/MS**	Protein profile of capacitated versus ejaculated human sperm	Identification of 29 proteins differentially expressed in capacitated sperm, swim-up selected capacitated sperm and ejaculated sperm	2009 (89)
**2DE-DIGE, in-gel digestion MALDI-TOF/TOF-MS**	Identification of diabetes- and obesity-associated proteomic changes in human spermatozoa	Identification of seven and nine proteins associated with type-1 diabetes and obesity, respectively	2009 (90)
**2DE, in-gel digestion MALDI-MS/MS**	To understand the molecular basis of sperm motility using asthenozoospermic sperm versus controls	Identification of eight proteins showing different abundance	2010 (91)
**2DE-DIGE, in-gel digestion MALDI-TOF/TOF MS and LC-MS**	The sperm protein profile was compared between fertile and oligoasthenozoospermic men	Identification of four proteins showing different abundance	2011 (92)
**2DE-DIGE, in gel digestion MALDI-TOF/TOF MS and LC-MS/MS**	To unveil disease-associated proteomic changes potentially affecting male fertility, the proteomes of sperm cells from type-1 diabetic, type-2 diabetic, non-diabetic obese and clinically healthy individuals, comparatively analyzed	Identification of 12 (type-1 diabetic), 71 (type-2 diabetic) and 13 (nondiabetic obese) proteins showing different abundance. Eppin protein complex components increased in sperm from the three groups of patients	2011 (93)
**2DE, in-gel digestion MALDI-TOF MS**	To screen and investigate the differentially expressed proteins in the sperm of infertile patients, whose sperm parameters met the WHO guidelines	Identification of 24 proteins showing different abundance	2012 (94)
**Washing IMAC with a phosphoprotein enrichment kit, in-solution digestion LC-MS**	To investigates the phosphoproteins involved in sperm motility in an attempt to identify the key pathways regulating sperm motility and likely to be altered in spermatozoa of asthenozoospermic individuals	Identification of 66 differentially regulated phosphoproteins	2012 (95)
**In-solution digestion LC-MS/MS**	To compare the proteomic profiles of spermatozoa exhibiting an impaired capacity for sperm-egg recognition with normal cells	Identification of seven proteins showing different abundance in sperm unable to bind to the ZP	2012 (96)
**2DE-DIGE, in-gel digestion LC-MS**	Spermatozoa suspensions from ROS+ and ROS− groups analyzed	Identification of 31 protein spots showing different abundance (25 increased and 6 decreased)	2013 (80)
**2DE-DIGE, in-gel digestion MALFI-TOF/TOF MS**	To explore the differentially expressed proteins in normal sperm motility and idiopathic asthenozoospermia	Identification of 15 proteins showing different abundance	2013 (97)
**SDS-PAGE in-gel digestion LC-MS/MS**	To screen for associations between sperm protein profiles and sperm concentration, motility, and DNA fragmentation index	Identification of 4 protein groups that correlate with DNA fragmentation and/or motility	2013 (98)
**SDS-PAGE in-gel digestion LC-MS/MS**	Analysis of the human sperm profile with high sperm counts	Significant inter-individual variation in head sperm protein profiles	2013 (99)
**In-solution digestion LC-MS/MS**	To examine if elevated levels of reactive oxygen species cause an alteration in the proteomic profile of spermatozoa	Identification of 15 proteins showing different abundance (10 increased and five decreased abundance)	2013 (81)
**In-solution digestion 2D LC-MS/MS**	The human sperm proteome profile of law and high DNA fragmentation of normozoospermic men	Identification of 71 (low DNA fragmentation) and 23 (high DNA fragmentation) proteins showing different abundance	2013 (100)
**In-solution digestion, 6-plex TMT labeling, LC-MS/MS**	Proteomic profiling of the human spermatozoa following successful or unsuccessful pregnancy via assisted reproductive technology (ART)	Identification of 21 proteins showing different abundance	2013 (101)
**2-plex TMT labeling, SDS-PAGE in gel digestion LC-MS/MS**	Normozoospermic sperm proteome samples with different IVF outcomes (pregnancy versus no pregnancy) compared	Identification of 64 proteins showing different abundance	2014 (102)
**2D-DIGE, in-gel digestion MALDI-TOF MS**	Proteomics profiling of the human sperm cryopreservation	27 proteins showing different abundance	2014 (103)
**2D-DIGE, in-gel digestion MALDI-TOF MS/MS**	Proteome analysis of sperm samples collected by swim-up from control and acute epididymitis patients analyzed	35 proteins showing different abundance	2014 (104)
**2D-DIGE in-gel digestion MALDI-TOF MS or LC-MS/MS**	Proteomic profiles of spermatozoa in patients with a complete failure of fertilization and no spermatozoa bound to the zona pellucida compared with those of controls	Identification of 12 proteins showing different abundance	2014 (105)
**SDS-PAGE in-gel digestion LC-MS/MS**	To investigate the human sperm proteome and its relation to blastocyst development and reproductive success	Identification of 49 proteins showing different abundance in sperm resulting in bad embryo development	2014 (106)
**In-solution digestion LC-MS/MS**	Differential proteomic analysis was performed on spermatozoa from both obesity-associated asthenozoospermia and clinically healthy individuals	Identification of 127 proteins showing different abundance	2014 (107)
**6-plex TMT labeling, SDS-PAGE in gel digestion LC-MS/MS**	The proteomics study was based on a comparison between sperm samples differing in motility (asthenozoospermic versus normozoospermic) and comparison between sperm subpopulations of fractionated normozoospermic samples differing in motility (non-migrated versus migrated)	Similar proteomic alterations detected in asthenozoospermic and nonmigrated sperm	2014 (108)
**SDS-PAGE in-gel digestion LC-MS/MS**	Study on immature and mature ejaculated sperm from fertile men	98 of them showed an increasing trend in expression levels	2016 (109)

The primary studies of the human spermatozoa proteome which used the differential proteomics approaches were focused on failure in the *in vitro* fertilization (IVF) due to male factor (83, 87). The authors identified 32 proteins which could improve the understanding of IVF failure due to male factor. They used gel-based proteomics technology (2DE followed by MALDI-TOF-MS protein identification). More recently, two other studies have used gel-free applications of proteomics approaches (6-plex TMT labeling followed by LC-MS/MS) on the human sperm to dig deeper on understanding IVF failure due to male factor (101, 102). Altogether, the mentioned studies have reported 85 deregulated proteins suggesting that epigenetic alterations may contribute to failure of assisted reproduction. Another interesting published study is based on frozen–thawed versus fresh human spermatozoa proteome that showed a malfunction of spermatozoon after cryopreservation (103).

On the other hand, several studies have focused on the asthenozoospermic patients. The importance of these patients is the high number of them and identification of proteins which are involved in the sperm motility. Furthermore, a sufficient amount of spermatozoa proteins can be easily extracted from asthenozoospermic sperm (84–86, 91, 95, 97, 107, 110).

Taken together, all deregulated identified proteins which have used MS-based proteomics technology shared protein involved in the cytoskeleton, metabolism or energy production (41).

Some studies have focused on reactive oxygen species (ROS) effect on the human spermatozoa. An imbalance in oxidative stress caused by a high generation of ROS by mitochondria has an effect on DNA of the human spermatozoa. Furthermore, it has an effect on the spermatozoa proteome (80, 81, 98, 100).

Very few studies have focused on the globozoospermic and oligoasthenozoospermic sperm. Both studies showed an altered proteome compared to fertile human sperm proteome (88, 92).

Two different studies have revealed the harmful consequence of the metabolic diseases including diabetes or obesity on the human sperm; however, the damaging effect on male fertility is not well identified at the molecular level. In their studies, they found the significant changes in the composition of the human sperm proteome (90, 93).

Finally, Cui et al. applied the proteomics to a relevant human fertility model and identified proteins which were critical for sperm maturation, motility and fertilization capacity (109).

## Conclusion

Great efforts have been done to explore the human proteome after identification of the human genome. Fifteen years after the first draft of the human genome, it is obvious today that the complexity of the human lays on the human proteome. A network of scientific collaboration has investigated human proteome mapping using advanced mass spectrometry-based proteomics. Regarding the proteome mapping of the human spermatozoa, the research is still in its infancy in spite of knowing the fact that male factor contributes 50% to infertility. In this review, the human proteome information was assessed with the specific focus on the human sperm proteome anno 2017. The most precise human protein database shows ∼21,931 proteins. Furthermore, some researchers have been able to identify on average 10,361 proteins from cell lines using advance mass spectrometry-based proteomics. However, the number of identified proteins from the human spermatozoa is limited to ∼6,500. This can be caused by either reaching mass-spectrometry current limitations or not reaching the depth of human spermatozoa proteome.

In order to go deeper in identification of the human proteome, the proteomics researchers have formed the international consortium for the Chromosome-Centric Human Proteome Project. Iran is leading the mapping of Y chromosome. Accordingly, by looking at the human Y chromosome-encoding proteins, it is clear that these proteins are low expressed in the human sperm. It is, furthermore, recognized that the human sperm proteins are also low expressed compared to other cells. However, with the development of mass spectrometry and miniaturization of sample preparations, it seems there is still work to do regarding identification and quantification of the human Y chromosome-encoded proteins.

To conclude, despite several publications that have focused on many comparative and functional sperm proteomic studies and providing putative biomarkers for male (in) fertility, some points are still unclear. The use of higher throughput techniques coupled to various up-to-date options for differential proteomics might provide further light toward knowledge of sperm (dys) functions at molecular level.
